# A Snapshot of United States Sarcoidosis Patients and their Perceived Disease Impact: Results of the Sarcoidosis Research Institute Survey

**DOI:** 10.1007/s00408-024-00761-8

**Published:** 2025-01-22

**Authors:** Ogugua Ndili Obi, Paula Yette Polite, Kenneth M. Fish, Robert DeLuca, Paul J. Feustel, Alexandra E. Mandis, Annetta M. Coleman, Marc A. Judson

**Affiliations:** 1 Division of Pulmonary, Critical Care, and Sleep Medicine, The Brody School of Medicine, East Carolina University, 600 Moye Blvd, Greenville, NC 27834 USA; 2 Sarcoidosis Research Institute, Memphis, TN 38111 USA; 3 Division of Pulmonary and Critical Care Medicine, Albany Medical College, 16 New Scotland Avenue, MC-91, Albany, NY 12208 USA; 4 Department of Neuroscience and Experimental Therapeutics, Albany Medical College, 16 New Scotland Avenue, MC-91, Albany, NY 12208 USA; 5 Independent Researcher, Cambridge, MA USA; 6 Independent Researcher, Albany, NY USA

**Keywords:** Sarcoidosis, United States (US), Shared decision-making (SDM), Health-related quality of life (HRQoL), Patient concerns, Patient perceptions, Patient priorities, Fatigue

## Abstract

**Purpose:**

The priorities and concerns of sarcoidosis patients in the United States (US) have not been well-described.

**Methods:**

A survey constructed by sarcoidosis patients and doctors was administered to US sarcoidosis patients. The survey queried patients concerning their demographics, disease state, disease impact on health and well-being, health care priorities and impressions of sarcoidosis care. Respondents were solicited via social media and networking with sarcoidosis clinicians.

**Results:**

1018 US sarcoidosis patients completed this survey. 65% were female, 63% White, 34% Black, and 87% > 45 years old. The most common organs involved were the lungs 87%, skin 30%, heart 25%, and eyes 25%. Household income was < $50 K in 31% and > $150 K in 14% of patients. There was a fairly even split between those living in urban (29%), suburban (42%), and rural (29%) environments. The patients’greatest concerns were fear of worsening disease, fear of sarcoidosis developing in more organs, and fear of sarcoidosis not improving. These were closely followed by concerns about poor health-related quality of life (HRQoL), inability to enjoy everyday activities, lack of medical research, disability from sarcoidosis, and pulmonary function status. Lack of physician knowledge and poor physician communication were ranked of lowest concern. Concerns about ineffective medications and cost of medical care were also ranked relatively low. Patients overwhelmingly considered information from their doctor as very useful.

**Conclusion:**

In this survey of over 1000 US sarcoidosis patients, their greatest concerns were fear of poor clinical outcomes. The patients were relatively less concerned about their doctors’ knowledge about sarcoidosis and poor physician communication. Although patients expressed significant concerns about poor HRQoL, not all domains of HRQoL were equally affected. US sarcoidosis patients rank concerns about disease progression higher than disease impact on HRQoL.

**Supplementary Information:**

The online version contains supplementary material available at 10.1007/s00408-024-00761-8.

## Introduction

Sarcoidosis is a chronic granulomatous pulmonary-predominant multi-organ systemic inflammatory disease [1]. The clinical manifestations of sarcoidosis, its severity and the response to treatment are highly variable and are affected by age, gender, race, ethnicity, geo-location, and socioeconomic status (SES) [2–6]. Though disease mortality is low (< 5%) [1] and over 50% of sarcoidosis patients have spontaneous disease remission, up to 50% may require treatment at some point for persistent disabling symptoms and 10**–**20% will need long-term therapy for chronic progressive disease [3, 7]. For most sarcoidosis patients, treatment decisions are based predominantly on health-related quality of life (HRQoL) issues [8, 9]. However, treatment can also be associated with significant toxicity which can impair HRQoL [10–13]. Consequently, the treatment of sarcoidosis requires a patient-centric shared decision-making (SDM) approach where the clinician incorporates patients’perceptions of the impact of disease on quality of life, the potential adverse effects of treatment, and overall well-being into treatment decisions [1, 12, 14, 15].

In a survey of sarcoidosis patients living in six European countries, improvements in HRQoL and functionality were regarded as more important treatment outcomes than improvement in objective measures including pulmonary function tests and imaging studies [9]. Similarly, a survey of sarcoidosis patients living in the Netherlands found that the disease significantly impacted the lives of both patients and their partners—sarcoidosis patients regularly felt misunderstood and had significant concerns about the lack of adequate information about their disease [16]. Finally, a survey of over 1000 sarcoidosis patients from Denmark, Germany, and the Netherlands found that most patients considered the disease burdensome [17]. Almost all patients (95%) were symptomatic (most commonly with fatigue, small fiber neuropathy (SFN), and pulmonary symptoms) yet up to 30% of patients had never been treated [17].

There are several potential reasons why these results from European sarcoidosis patients may not be reliably extrapolated to United States (US) sarcoidosis patients. First, the demographics of US sarcoidosis patients differs from those in Europe [2, 5]. Second, the disease characteristics of sarcoidosis vary between the United States and Europe [2]. Third, the structure of the US and European health care systems are vastly different [18, 19]. In addition, the US and Europe differ greatly in terms of economics, culture, and social structure. To date, no study has evaluated the perceptions, concerns, or perspectives of US sarcoidosis patients [20]. We surveyed over 1000 patients with sarcoidosis living in the US and attempted to quantify their concerns about the state of their disease, treatment, disease outcomes, and quality of life.

## Study Design and Methods

### Study Design

We conducted a nationwide survey of US sarcoidosis patients between March 1, 2023, and November 30, 2023.

### Development of the Survey Instrument

The survey instrument was constructed by sarcoidosis patients (PYP, AEM, AC) and physicians dedicated to the care of sarcoidosis patients (ONO, MAJ) using a web-based survey platform—*SurveyMonkey* (www.surveymonkey.com) [21]. Another sarcoidosis patient who also participated actively in the design of the survey (and other aspects of the project) deserving of authorship declined to be listed for privacy concerns. Construction of the survey instrument was a collaborative effort and involved several 45-to-60-min virtual meetings and extensive email communication over a 6-week period. At the first meeting, each team member identified concerns they had as sarcoidosis patients or had gleaned from caring for sarcoidosis patients. Next, these concerns were grouped together in broad themes and the team brainstormed how to best present these concerns as concise questions. Ambiguous and duplicate questions were eliminated, and common themes were consolidated. Our overarching goals were to (1) keep the survey brief (completion within 5-min) to optimize response rates and (2) ensure that the concerns of the patients were clearly reflected in the final instrument. Prior to finalizing our survey instrument, we tested the survey in 12 sarcoidosis patients in a sarcoidosis clinic (Albany Medical Center) and obtained additional feedback regarding the quality of the questions, the length of time to complete the survey, and the breadth of concerns addressed. This feedback was incorporated into the final draft of the survey which was then circulated to each team member for approval. Subsequently, the survey was placed on the *Sarcoidosis Research Institute* web page (https://www.sarcoidosisri.org) [22]. The final survey queried sarcoidosis patients concerning their demographics, socioeconomic status, level of education, place of residence, organ involvement, aspects of sarcoidosis treatments, symptoms, and levels of concern about 23 issues concerning sarcoidosis (eFigure 1). The survey instrument was approved for use as an anonymous public survey by the institutional review board (IRB) of Albany Medical College (IRB #: 6648).

### Solicitation of Survey Respondents

Respondents were solicited via social media, by networking with sarcoidosis clinicians, and by contacting sarcoidosis patient groups. Team members placed links to the survey on various social media platforms including Facebook, LinkedIn, Reddit, and X (formerly Twitter). Sarcoidosis clinicians were solicited in various ways—by email blasts to known sarcoidosis physicians in charge of sarcoidosis clinics and centers of excellence across the US, by a sarcoidosis Listserv developed and maintained by the Americas Association of Sarcoidosis and Other Granulomatous Diseases (AASOG), and by word of mouth. We also placed links to the survey on participating clinic websites and took out a month-long advertisement on *Sarcoidosis News (*https://sarcoidosisnews.com*)* [23], a web-based sarcoidosis newsletter with broad readership by US sarcoidosis patients. Due to our study design (nationwide survey), no sample size calculations were performed. However, we aimed to obtain at least 1,000 responses.

### Study Population

To ensure that only sarcoidosis patients completed the questionnaire, a preliminary survey question identified whether the responder had sarcoidosis, or had a friend, spouse/partner, or family member with sarcoidosis. An option for none of the above was also included. Next, we identified whether respondents were US residents and their state of residence. Though all self-identified sarcoidosis patients (or friends/relatives of sarcoidosis patients) were allowed to complete the survey, only US resident sarcoidosis patients who completed survey questions were analyzed.

### Data and Statistical Analysis

Categorical data is presented as frequencies and percentages. For the 23 sarcoidosis issues, the ordinal responses for the level of concern were coded as “none at all”: 0; “a little”: 1, “a moderate amount”: 2, “a lot”: 3, and “a great deal”: 4. We analyzed these data in two ways. First, we calculated the mean and standard deviation for the level of concern about each issue to enable comparisons among the responses. Second, to determine the relative importance of each of these issues in individual patients, we also calculated the response score difference for each issue from the average score of of the level of concern for all 23 issues for each patient. Analysis of variance was conducted with pairwise comparisons by Tukey’s Honest Significant Difference (HSD) test. Relative rankings of patients in North Carolina (NC), New York (NY), South Caolina (SC), and Colorado (CO) were compared to rankings in other states by correlation analysis. Minitab® 19 and R 4.3.0 statistical software were used.

## Results

A total of 1189 individuals completed the survey between March 1, 2023, and November 30, 2023. Ninety-seven percent (1097 respondents) had sarcoidosis. A total of 3% (37 respondents) identified themselves as either a friend, spouse/partner, or family member of a sarcoidosis patient and were not included in the analysis. An additional 55 respondents did not specify what relationship they had with sarcoidosis (question #8, eFigure 1) and were not included in the analysis. Of the 1097 patients identifying themselves as sarcoidosis patients, we excluded patients who were not US residents (*n* = 61) or who were missing residence state data (*n* = 18) for a final sample size of 1018 US sarcoidosis patients who were analyzed.

**Fig. 1:**
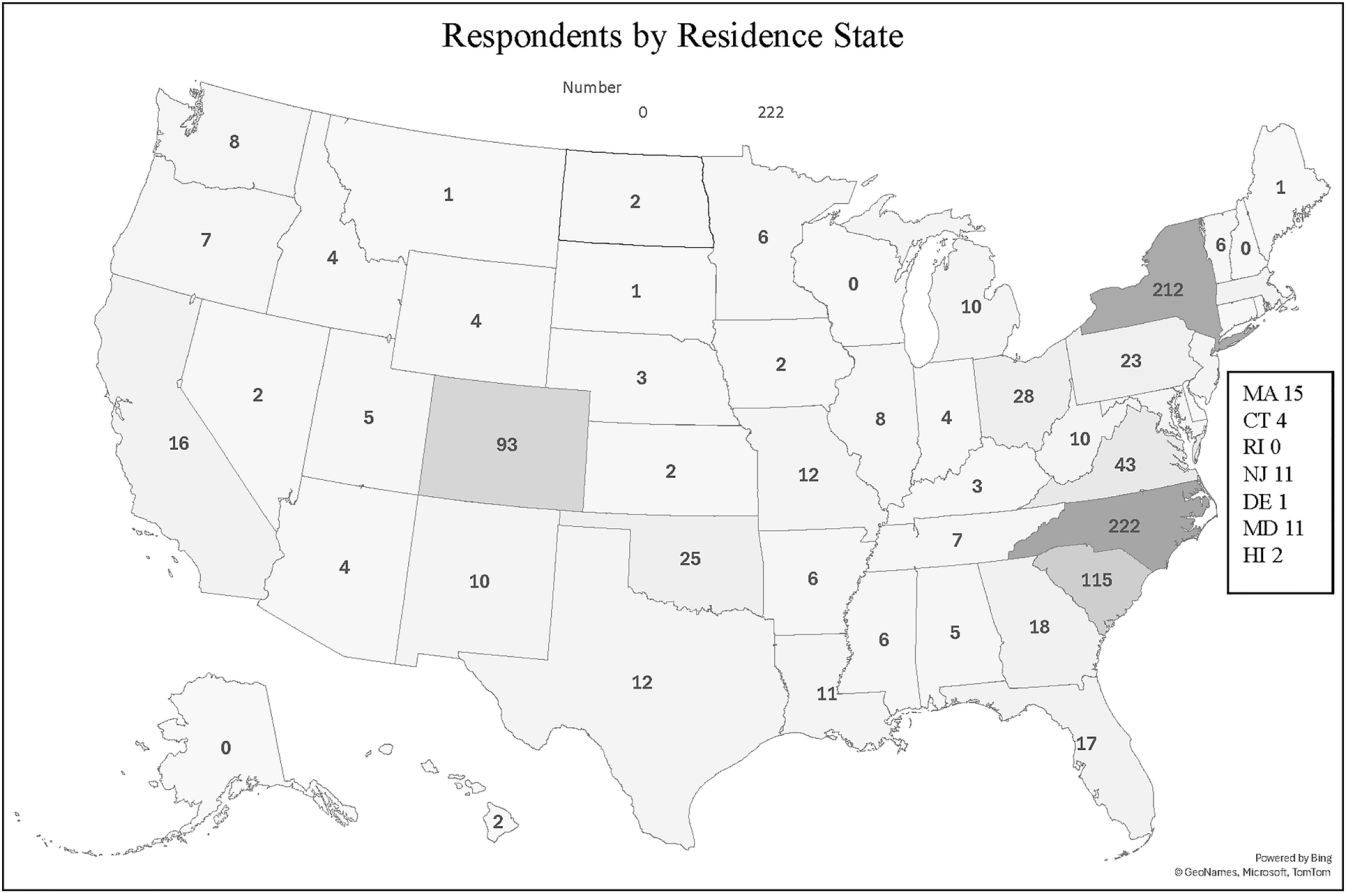
Heat Map of the United States Showing Where Respondents Live. Forty-seven states were represented in our study however, 60% of survey respondents were from four (4) states-North Carolina (21%), New York (20%), South Carolina (11%), and Colorado (9%). Other states that contributed 20 or more patients include Ohio (3%), Virginia (4%), Oklahoma (2%), and Pennsylvania (2%)

Patient demographics are presented in Table 1. Eighty-seven percent (87%) of our study population were 45 years or older, 65% were female, 34% self-identified as Black or African American and 63% as White. More than one-third (36%) of patients had a household income < $50,000/year, 17% had a household income > $150,000/year, and over 50% had a college degree or higher (Table 1). Figure 1 shows that although there was a wide representation of patients across the US (47 states represented), more than sixty percent (63% or 642/1018) were from 4 states (North Carolina, New York, South Carolina, and Colorado). This disproportionate number of respondents from these states probably reflects major sarcoidosis centers in these states where we are aware that respondents were actively recruited to participate. There was a fairly even split between those living in urban (29%), suburban (42%), and rural (29%) areas (Table 1).

**Table 1:** Baseline Demographics and Patient Characteristics

Age Range (number of responses, *N* = 1016)	*n*	(%)
18–24	1	(0.1%)
25–34	34	(3%)
35–44	101	(10%)
45–54	222	(22%)
55–64	337	(33%)
65–74	254	(25%)
> 74	67	(7%)
Sex (*N* = 1008)		
Female	655	(65%)
Male	353	(35%)
Race (*N* = 1018)		
White	646	(63%)
Black or African American	344	(34%)
Hispanic or Latino	22	(2%)
Asian	7	(1%)
Native American or Alaskan	13	(1%)
Household Salary (*N* = 1010)		
Prefer not to answer	165	
< $15 k	76	(9%)
$15 k—$50 k	229	(27%)
$50 k—$100 k	256	(30%)
$100 k—$150 k	143	(17%)
> $150 k	141	(17%)
Highest Level of Education (*N* = 1013)		
Prefer not to answer	14	
Less than High School	34	(3%)
High School	170	(17%)
Some College	231	(23%)
College	312	(31%)
Some Graduate School	44	(4%)
Graduate School	208	(21%)
Describe where you Live (*N* = 1018)		
Urban/City	293	(29%)
Rural	300	(29%)
Suburban	425	(42%)
Residence (*N* = 1018)		
NY, NC, SC, CO	642	(63%)
Other States	376	(37%)

### Disease Characteristics and Symptoms

Table 2 displays the patients’disease characteristics and their symptoms. The lungs were the most commonly involved organ (87% of patients) followed by the skin (30%), the heart (25%), and the eyes (25%). Sixty-two percent (631/1018) of patients had multi-organ involvement. Most patients (80%) had sarcoidosis for > 2 years. Seventy-one percent (706/999) considered their disease to be active and 65% (655/1013) were currently on therapy. Patients rated the degree to which they had experienced several disease-related symptoms on a 5-point Likert scale (not at all (0), a little (1), a moderate amount (2), a lot (3), or a great deal (4)). Fatigue was the symptom most frequently (42%) experienced “a lot” or “a great deal” followed by depression, emotional distress or mental health (34%), chronic pain (28%), and shortness of breath (23%).

**Table 2:** Disease Characteristics and Patient Symptoms

Organs affected (number of responses, *N* = 1018)					*n*	%	
		Lungs			881	(87%)	
		Heart			252	(25%)	
		Skin			303	(30%)	
		Brain or Nervous System			147	(14%)	
		Eyes			252	(25%)	
		Other Organs			284	(28%)	
Number of organs affected (*N* = 1018)							
		0		3		(0.3%)	
		1		384		(38%)	
		2		325		(32%)	
		3		190		(19%)	
		4		77		(8%)	
		5		27		(3%)	
		6		12		(1%)	
Disease Duration (*N* = 1012)							
	Don’t know		21				(2%)
	< 1 year		83				(8%)
	1–2 years		96				(9%)
	> 2 years		812				(80%)
Current Disease Status (*N* = 999)							
	Not active		293				(29%)
	Active		706				(71%)
Current Treatment (*N* = 1013)							
	None		358				(35%)
	On medication		655				(65%)
Symptom with a severity of “A Lot” or “A Great Deal”							
	Fatigue (*N* = 992)		415				(42%)
	Chronic pain (*N* = 975)		275				(28%)
	SOB (*N* = 986)		230				(23%)
	Cough (*N* = 990)		182				(18%)
	Depression (*N* = 982)		134				(14%)
	Emotional distress (*N* = 979)		118				(12%)
	Eye/vision problems (*N* = 972)		108				(11%)
	Heart palpitations (*N* = 974)		89				(9%)
	Mental health issue (*N* = 979)		81				(8%)
	Skin rash (*N* = 973)		72				(7%)
	Chest pain (*N* = 972)		65				(7%)

### Source of Information about Sarcoidosis

Table 3 shows the patients’assessment of the quality of the information they receive about sarcoidosis. Most patients (69%) rated the information that they received from their doctors about sarcoidosis as very useful or extremely useful, followed by 33% who considered information obtained from the internet very useful or extremely useful. (Table 3).

**Table 3:** Quality of Information Received from Various Sources

	Not Applicable	Not Useful at All	Not So Useful	Somewhat Useful	Very Useful	Extremely Useful
My doctor (*N* = 978)	1%	4%	6%	20%	25%	44%
The internet (*N* = 967)	7%	4%	9%	47%	22%	11%
Printed media and publications (*N* = 973)	23%	9%	15%	35%	12%	6%
Sarcoidosis in-person group meeting (*N* = 966)	64%	10%	5%	9%	6%	5%
Other sarcoidosis patients (*N* = 968)	48%	8%	6%	16%	11%	10%

### Patient Concerns

Table 4 shows the patients’ level of concern for 23 sarcoidosis issues. More than 87% of sarcoidosis patients reported their level of concern to all 23 issues. The areas of most concern involved worsening disease or fear of sarcoidosis not improving. Poor physician knowledge and ineffective communication with the doctor were of relatively little concern.

**Table 4:** Sarcoidosis Patient Concerns

	None at all (0)	A Little (1)	Moderate Amount (2)	A Lot (3)	A Great Deal (4)	Mean Score	SD	Number Responding (N)
Difficulties in making the diagnosis of sarcoidosis	35%	22%	18%	12%	12%	1.44	1.39	975
Lack of my doctor’s knowledge of sarcoidosis	61%	11%	10%	7%	10%	0.94	1.38	974
Ineffective communication with my doctor	63%	15%	8%	6%	8%	0.80	1.27	975
Side effects from medication	31%	21%	21%	13%	14%	1.59	1.41	965
Ineffective medications	41%	22%	17%	9%	11%	1.28	1.36	954
Adequate health insurance/cost of medical care	39%	21%	16%	10%	15%	1.41	1.45	967
Lack of information available about sarcoidosis	30%	21%	18%	14%	17%	1.66	1.45	974
Lack of medical research concerning sarcoidosis	22%	19%	22%	16%	22%	1.96	1.45	970
Fear of worsening disease	6%	24%	25%	19%	26%	2.33	1.26	976
Fear of developing sarcoidosis in more organs	8%	25%	24%	17%	27%	2.31	1.31	980
Fear of sarcoidosis not improving	11%	24%	23%	18%	24%	2.20	1.34	974
Death from sarcoidosis	23%	26%	16%	11%	23%	1.85	1.49	974
My pulmonary function status	15%	28%	25%	14%	17%	1.90	1.31	971
My X-ray or CT findings	21%	29%	24%	12%	14%	1.69	1.31	966
Risk of getting COVID infection	28%	24%	20%	12%	16%	1.64	1.42	973
Disability from sarcoidosis	20%	25%	18%	13%	24%	1.95	1.46	969
Poor quality of life from sarcoidosis	16%	25%	20%	16%	22%	2.02	1.39	979
Unable to enjoy everyday activities	18%	26%	19%	16%	21%	1.97	1.41	977
Unable to enjoy free time	24%	28%	16%	14%	19%	1.76	1.44	970
Poor relationships as a result of sarcoidosis	42%	23%	13%	9%	13%	1.26	1.41	976
Lack of compassion and understanding of my illness by others	35%	23%	17%	10%	15%	1.48	1.44	975
Embarrassment from having sarcoidosis	60%	17%	8%	7%	7%	0.83	1.26	973
Work performance adversely impacted by sarcoidosis	34%	21%	15%	10%	20%	1.61	1.52	972

**Fig. 2:**
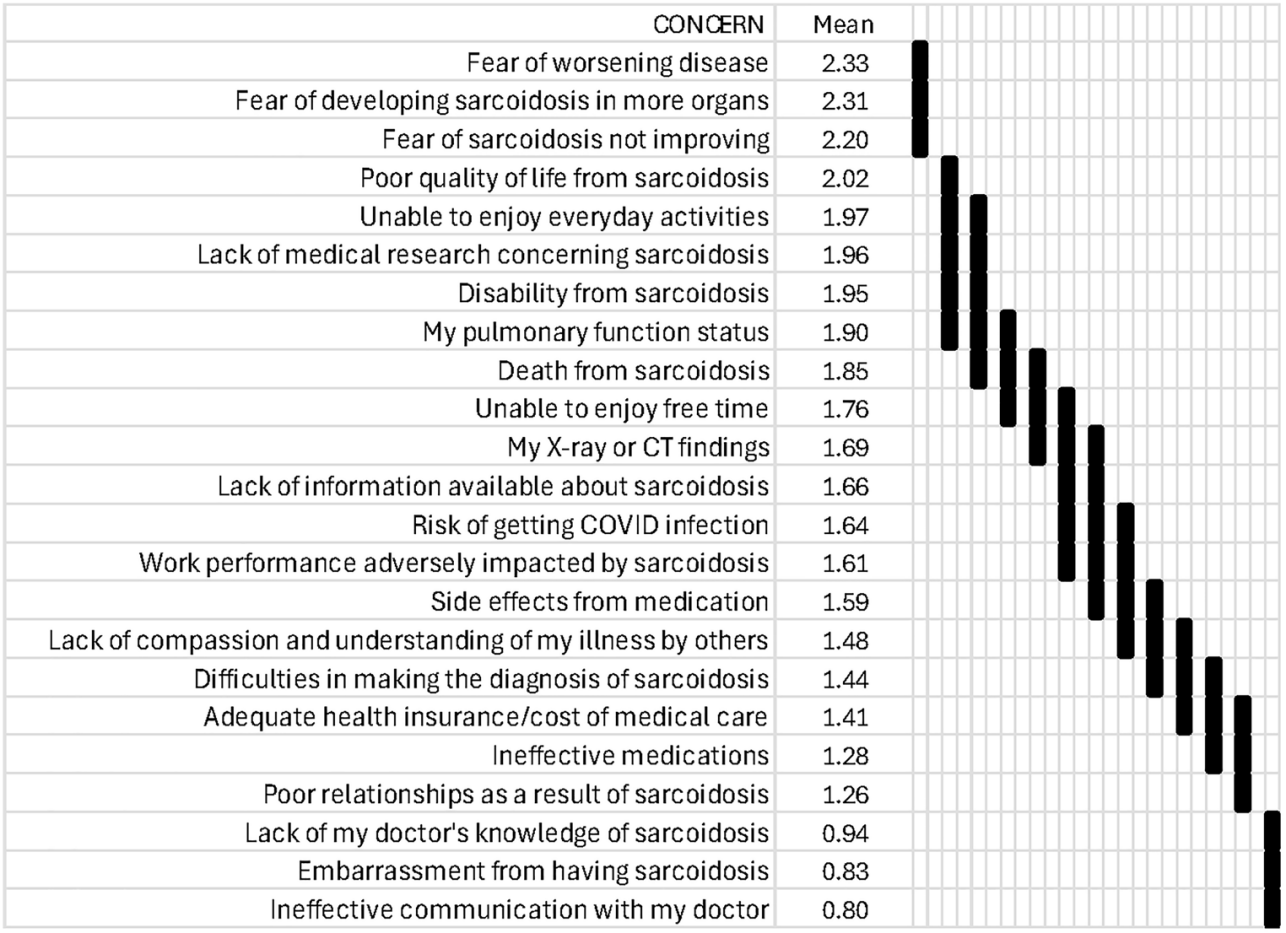
Ranking of the mean level of concern for each of the 23 sarcoidosis issues from highest to lowest. Means represent coded levels of concern where 0 = None, 1 = a little, 2 = moderate, 3 = a lot, 4 = a great deal. Vertical bars on the right connect issues with levels of concern that are not statistically significantly different from one another by ANOVA followed by Tukey’s multiple comparison test (applied to a subject's responses expressed as the difference from their average level of concern). For example, level of concern regarding “lack of medical research concerning sarcoidosis” is not significantly different from “Unable to enjoy everyday activities,” “disability from sarcoidosis,” “pulmonary function status,” and “death from sarcoidosis,” but is significantly less concerning than the first three listed fears and more concerning than “unable to enjoy free time” and all those listed below “unable to enjoy free time.” Minitab® 19 statistical software was used with significance assessed at *p* < 0.05

**Fig. 3:**
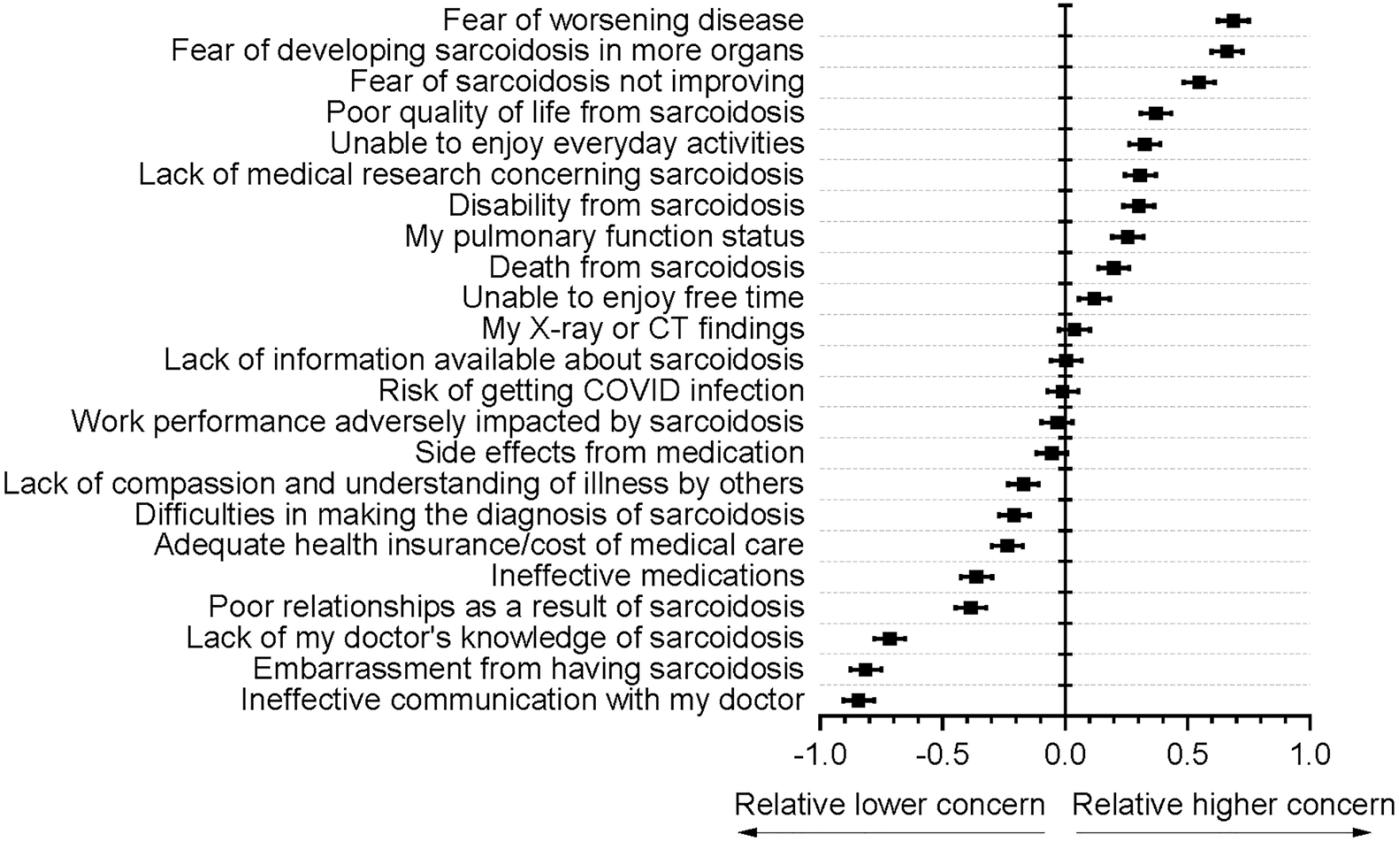
Relative Level of Concern of Each Individual Patient. Figure shows mean (solid square) with horizonal bars indicating 95% confidence intervals for the mean for 954 to 980 responses relative to each respondent’s average level of concern. In order to determine the relative importance of each of these concerns in individual patients, we calculated the response score difference for each concern from the average score of all concerns for each patient and these were ranked as shown in the figure

Figure 2 shows the 23 issues ranked by level of concern. Using each patient’s relative level of concern yielded 13 different levels of concern among the 23 issues with issues that presented levels of concern that were not statistically different from one another, connected by the vertical bars on the right. Figure 3 displays the mean and standard error of the mean of each patient’s level of concern relative to their mean level of concern across all 23 issues. The rank by absolute concern scores in Fig. 2 was identical to the relative concern rankings of Fig. 3. The top-ranked issues involved 4 central themes: fear of progressive or unresolving disease, poor HRQoL from sarcoidosis, lack of medical research concerning sarcoidosis, and disability from sarcoidosis. Contrary to prior studies, [9] concerns about pulmonary functional status were considered equal in importance to HRQoL and ranked similarly. Surprisingly, adequate health insurance, the cost of medical care, ineffective medications, and medication side effects were of relatively lower concern.

Comparing responses from participants in the four states with the highest number of respondents (NC, NY, SC, CO) to those from respondents in the other 46 states, the level of concern was significantly higher for all 23 issues in the latter group, with an average difference in the level of concern score of 0.528 points (SD = 0.195). The largest differences in concern score were observed in “lack of medical research” (0.91 points higher in the other 46 states), “lack of doctors knowledge” (0.87 points higher in the other 46 states), and “lack of knowledge about sarcoidosis” (0.83 points higher in the other 46 states) (Table 5). There were also some differences in the ranking of the 23 issues between patients in the four states with the highest number of respondents compared to respondents in the other 46 states. However, the rankings of sarcoidosis patients living in the four states versus the other 46 states were highly correlated (spearman’s correlation 0.897, 95% CI 0.735 to 0.962) and the issues of most concern were ranked similarly by both groups of patients (Table 6 and supplemental eFigure 2).

**Table 5:** Comparison of the mean values on a 5-point Lickert scale (0-4) for each of the 23 sarcoidosis issues between patients residing in North Carolina (NC), New York (NY), South Carolina (SC) and Colorado compared to those residing in the other 46 states

	NC NY SC CO			Other states			
Factor	N	Mean score	StDev	N	Mean score	StDev	Difference in mean scores
Lack of medical research concerning sarcoidosis	619	1.63	1.42	351	2.54	1.31	0.910
Lack of my doctor’s knowledge of sarcoidosis	619	0.62	1.21	355	1.49	1.49	0.873
Lack of information available about sarcoidosis	621	1.36	1.38	353	2.18	1.43	0.825
Side effects from medication	613	1.32	1.35	352	2.05	1.38	0.727
Ineffective medications	605	1.02	1.30	349	1.72	1.37	0.696
Ineffective communication with my doctor	619	0.56	1.12	356	1.22	1.40	0.660
Fear of developing sarcoidosis in more organs	623	2.10	1.31	357	2.68	1.21	0.584
Difficulties in making the diagnosis of sarcoidosis	620	1.23	1.33	355	1.81	1.43	0.584
Lack of compassion and understanding of my illness by others	620	1.27	1.39	355	1.83	1.46	0.560
Poor quality of life from sarcoidosis	622	1.82	1.39	357	2.37	1.33	0.545
Fear of sarcoidosis not improving	620	2.01	1.33	354	2.54	1.29	0.536
Unable to enjoy everyday activities	622	1.78	1.40	355	2.31	1.35	0.529
Fear of worsening disease	622	2.15	1.27	354	2.65	1.20	0.495
Work performance adversely impacted by sarcoidosis	620	1.44	1.49	352	1.92	1.53	0.479
Disability from sarcoidosis	616	1.78	1.43	353	2.25	1.46	0.476
Poor relationships as a result of sarcoidosis	622	1.09	1.33	354	1.56	1.50	0.469
Unable to enjoy free time	616	1.60	1.42	354	2.05	1.43	0.453
Death from sarcoidosis	618	1.70	1.50	356	2.12	1.44	0.422
Adequate health insurance/cost of medical care	615	1.27	1.42	352	1.66	1.47	0.383
My X-ray or CT findings	613	1.59	1.31	353	1.86	1.30	0.271
Embarrassment from having sarcoidosis	618	0.74	1.20	355	1.00	1.35	0.258
My pulmonary function status	616	1.82	1.31	355	2.03	1.31	0.209
Risk of getting COVID infection	619	1.57	1.38	354	1.77	1.49	0.203
				Average difference			0.528
				Standard Deviation			0.195

**Table 6:** For each of the 23 sarcoidosis issues, the comparison of the rank of each patient's level of concern relative to their average level of concern for all of the 23 issues (see figure 3) in patients residing in North Carolina (NC), New York (NY), South Carolina (SC), and Colorado (CO) compared to those residing in the other 46 states

	NC NY SC CO				Other States				
Factor	N	Mean	StDev	Rank	N	Mean	StDev	Rank	Difference in Rank
Fear of developing sarcoidosis in more organs	623	0.641	0.766	2	357	0.696	0.793	1	1
Fear of worsening disease	622	0.699	0.726	1	354	0.667	0.758	2	−1
Fear of sarcoidosis not improving	620	0.546	0.783	3	354	0.552	0.834	3	0
Lack of medical research concerning sarcoidosis	619	0.169	1.106	9	351	0.551	1.037	4	5
Poor quality of life from sarcoidosis	622	0.367	0.800	5	357	0.379	0.768	5	0
Unable to enjoy everyday activities	622	0.326	0.839	6	355	0.325	0.833	6	0
Disability from sarcoidosis	616	0.321	0.901	7	353	0.265	0.975	7	0
Lack of information available about sarcoidosis	621	− 0.102	1.059	14	353	0.192	1.112	8	6
Death from sarcoidosis	618	0.236	1.006	8	356	0.136	1.031	9	−1
Side effects from medication	613	− 0.127	1.112	15	352	0.074	1.214	10	5
Unable to enjoy free time	616	0.147	0.852	10	354	0.070	0.880	11	−1
My pulmonary function status	616	0.371	0.902	4	355	0.056	1.054	12	−8
Work performance adversely impacted by sarcoidosis	620	− 0.019	1.102	13	352	− 0.057	1.198	13	0
My X-ray or CT findings	613	0.128	0.854	11	353	− 0.119	0.960	14	−3
Lack of compassion and understanding of my illness by others	620	− 0.181	0.958	16	355	− 0.153	1.000	15	1
Difficulties in making the diagnosis of sarcoidosis	620	− 0.229	1.146	18	355	− 0.170	1.296	16	2
Risk of getting COVID infection	619	0.108	1.100	12	354	− 0.215	1.213	17	−5
Ineffective medications	605	− 0.422	1.004	20	349	− 0.257	1.084	18	2
Adequate health insurance/cost of medical care	615	− 0.184	1.195	17	352	− 0.328	1.212	19	−2
Poor relationships as a result of sarcoidosis	622	− 0.365	0.886	19	354	− 0.420	1.014	20	−1
Lack of my doctor’s knowledge of sarcoidosis	619	− 0.840	1.084	22	355	− 0.500	1.263	21	1
Ineffective communication with my doctor	619	− 0.890	1.052	23	356	− 0.764	1.170	22	1
Embarrassment from having sarcoidosis	618	− 0.718	1.007	21	355	− 0.982	1.087	23	−2

## Discussion

Our survey of over 1000 US sarcoidosis patients found that there was significant concern about many aspects of their disease. The patients’greatest concerns were about poor clinical outcomes such as worsening disease, developing sarcoidosis in more organs, and fear of sarcoidosis not improving. US sarcoidosis patients also expressed a high level of concern about poor HRQoL, inability to enjoy everyday activities, disability from disease, pulmonary function status and lack of medical research concerning sarcoidosis. Interestingly, patients considered concerns about lack of physician knowledge and poor physician communication to be of relatively lower importance. Adequate health insurance, the cost of medical care, and ineffective medications were also ranked lower than expected. Remarkably, although concern about poor HRQoL was ranked highly, not all domains of HRQoL were equally affected [12, 24]. Patients ranked the inability to enjoy everyday activities and free time (physical functioning) higher than social health domains such as embarrassment about sarcoidosis, poor relationships, or lack of understanding from others as a result disease. Our study results are robust in that we analyzed data in two different ways and observed an identical ranking of all 23 sarcoidosis issues.

These survey results suggest that although sarcoidosis patients in the US and Europe were similar in their level of concern about several sarcoidosis issues, there were also noteworthy differences [9, 16, 17, 20]. Similar to European sarcoidosis patients, fatigue was the most distressing symptom [16, 17]. Depression, emotional distress and mental health issues were also similarly common and equally distressing, as was chronic pain [16, 17]. In contradistinction, although both US and European sarcoidosis patients were very concerned about HRQoL issues, this was the prominent area of concern for European sarcoidosis patients whereas fear of worse disease outcomes was of primary importance to US sarcoidosis patients [9, 16, 20]. Notably, our study also revealed that although sarcoidosis affects HRQoL as a global construct, not all domains are equally affected. Patients ranked concerns about inability to enjoy everyday activities and free time (physical functioning) much higher than embarrassment, poor relationships, or impaired work-performance. Determining what aspects of HRQoL are most concerning to sarcoidosis patients may improve SDM and patient adherence to treatment regimens [25].

Unlike a large survey of European sarcoidosis patients [9], we found that US sarcoidosis patients ranked concerns about pulmonary function status very highly and equal in importance to HRQoL issues. The reasons for this are unclear but may be related to the sarcoidosis patients’source of medical information. It is plausible that this response may be a reflection of the health care provider’s viewpoint or perhaps a consequence of practice patterns that emphasize pulmonary function testing [13]. Nonetheless, both US and European sarcoidosis patients had relatively little concern about chest imaging findings [9].

It is important to emphasize that according to US sarcoidosis patients, the overwhelmingly most useful source of medical information was from their health care provider. This suggests that these patients are willing to participate in SDM if they are provided the opportunity by their caregiver. This finding also stresses the need for health care providers to remain well informed about sarcoidosis, especially because a significant proportion of sarcoidosis patients still feel inadequately educated about their disease [16], which has been shown to be a barrier to care, especially in sarcoidosis patients of low SES [26].

Some of our study findings were unexpected. US sarcoidosis patients completing our survey ranked lack of their doctors’knowledge and ineffective physician communication of low importance. In contradistinction, Harper and colleagues found that provider knowledge gaps and poor patient-provider communication emerged as highly significant barriers to care in a sarcoidosis patient focus group study conducted at a tertiary referral center [26]. We believe that the difference in findings between our study and that of Harper and colleagues has two likely explanations. First, the two studies asked different questions: ours concerned patient perceived disease impact, whereas the previous study focused on patient perceived barriers to care [26]. Second, 60 percent (60%) of our respondents were likely cared for at sarcoidosis centers of excellence, as they lived in the 4 states where we suspect sarcoidosis centers of excellence actively recruited participants. In fact, all 23 sarcoidosis issues were uniformly of less concern in the patients living in the 4 states where sarcoidosis centers of excellence actively recruited participation than in the other states represented in our study (Table 5). However, the relative concern of these 23 issues was extremely similar between these two groups (*r* = 0.897). Therefore, the priority of these concerns was almost identical in both these patient groups, suggesting that this relative level of concern is a universal finding.

We also found that adequate health insurance and cost of medical care were not a major concern for our patients. This likely reflects the relatively high SES of this cohort. It has been shown that sarcoidosis patients with a lower SES experience barriers to care because of medical care costs and inadequate health insurance [26]. Ineffective medications and medication side effects were also not a major concern. As we did not capture information concerning the patients’sarcoidosis medications or the dosages, we cannot ascertain if this finding relates to the percentage of sarcoidosis patients not receiving medications or to patients receiving a sarcoidosis treatment regimen that minimized drug toxicity, such as a corticosteroid-sparing or corticosteroid-replacing approach. We also cannot exclude the possibility that the corticosteroid-sparing agents that are currently available are adequate and that the need for alternative medications may not be as great an unmet need as currently thought. Nonetheless, the need for increased medical research in sarcoidosis was ranked very highly suggesting significant patient dissatisfaction with current sarcoidosis care.

Finally, concern about death from sarcoidosis was ranked highly. This may reflect that a high proportion of our patients had high-risk sarcoidosis phenotypes (cardiac and neurosarcoidosis) where the risk of death is significant [2, 5, 6]. A survey of European sarcoidosis patients also ranked survival as a major concern [9] although no data were collected on disease phenotypes. Future studies may need to evaluate patients’perceptions about death from sarcoidosis in relation to their disease severity and phenotype.

Our study has several limitations. First, patients self-reported their diagnosis of sarcoidosis, and we were unable to confirm this. In an attempt to address this limitation, we did specifically ask the respondents if they had sarcoidosis, were a relative or acquaintance of a sarcoidosis patient, or were in none of these categories. Secondly, organ involvement as well as medication use were also self-reported and may not be accurate. Thirdly, as with most survey studies, study participants were self-selected, and this may have induced biases in patient selection. An additional selection bias is that since this was an online survey, only patients with internet access and/or some social media presence could complete it. Finally, although we had broad representation of US sarcoidosis patients, 60% of study participants were from four states where a large percentage were likely cared for at four large sarcoidosis centers of excellence. These centers (often tertiary and/or referral institutions) are staffed by physicians well versed in sarcoidosis and are more likely to attract patients with more severe or chronic disease manifestations [27]. Consequently, our results may be biased towards sarcoidosis patients with more severe disease who also likely received more expert care. Remarkably, though we did note some slight differences in the ranking of sarcoidosis concerns of these patients compared to sarcoidosis concerns of patients residing in the other states, we found that the relative rankings of sarcoidosis concerns of patients in the top four states compared to patients from the rest of the country were very similar and highly correlated (*r* = 0.897).

## Conclusion

In conclusion, our survey found that US sarcoidosis patients have significant concerns about their disease. Similar to European sarcoidosis patients, US sarcoidosis patients expressed significant concerns about the effect of their disease on quality of life. Our patients were not equally concerned about different HRQoL domains. Patients were more concerned about HRQoL domains involved with physical functioning than those involving social health. In contrast to European sarcoidosis patients, US sarcoidosis patients were most concerned about fear of worse clinical outcomes, even more than HRQoL issues. Patients also had significant concern about improving sarcoidosis research. Lack of adequate information about sarcoidosis was a significant patient concern; however, patients did not consider lack of their doctors’knowledge or ineffective physician communication as significant concerns. Most US sarcoidosis patients consider information from their physician to be very useful, suggesting that they are willing to participate in SDM if they are provided this opportunity by their caregiver.

## Supplementary Information

Below is the link to the electronic supplementary material.

Supplementary file1 (TIFF 101 KB)

Supplementary file2 (TIFF 26 KB)

Supplementary file3 (TIFF 21 KB)

Supplementary file4 (TIFF 109 KB)

Supplementary file5 (TIFF 103 KB)

Supplementary file6 (TIFF 125 KB)

Supplementary file7 (TIFF 117 KB)

Supplementary file8 (TIFF 1522 KB)

## Data Availability

Data is provided within the manuscript and supplementary information files.

## Abbreviations

AASOG: Americas association of sarcoidosis and other granulomatous diseases (AASOG)
HRQoL: Health-related quality of life
SD: Standard deviation
SDM: Shared decision-making
SES: Socioeconomic status
SFN: Small fiber neuropathy
CO: Colorado
NC: North Carolina
NY: New York
SC: South Carolina
US: United States
