# Supplementary figures and images for: A Snapshot of United States Sarcoidosis Patients and their Perceived Disease Impact: Results of the Sarcoidosis Research Institute Survey
Source: Lung. 2025 Jan 22;203(1):31. doi: 10.1007/s00408-024-00761-8 (PMC11754370; doi:10.1007/s00408-024-00761-8)

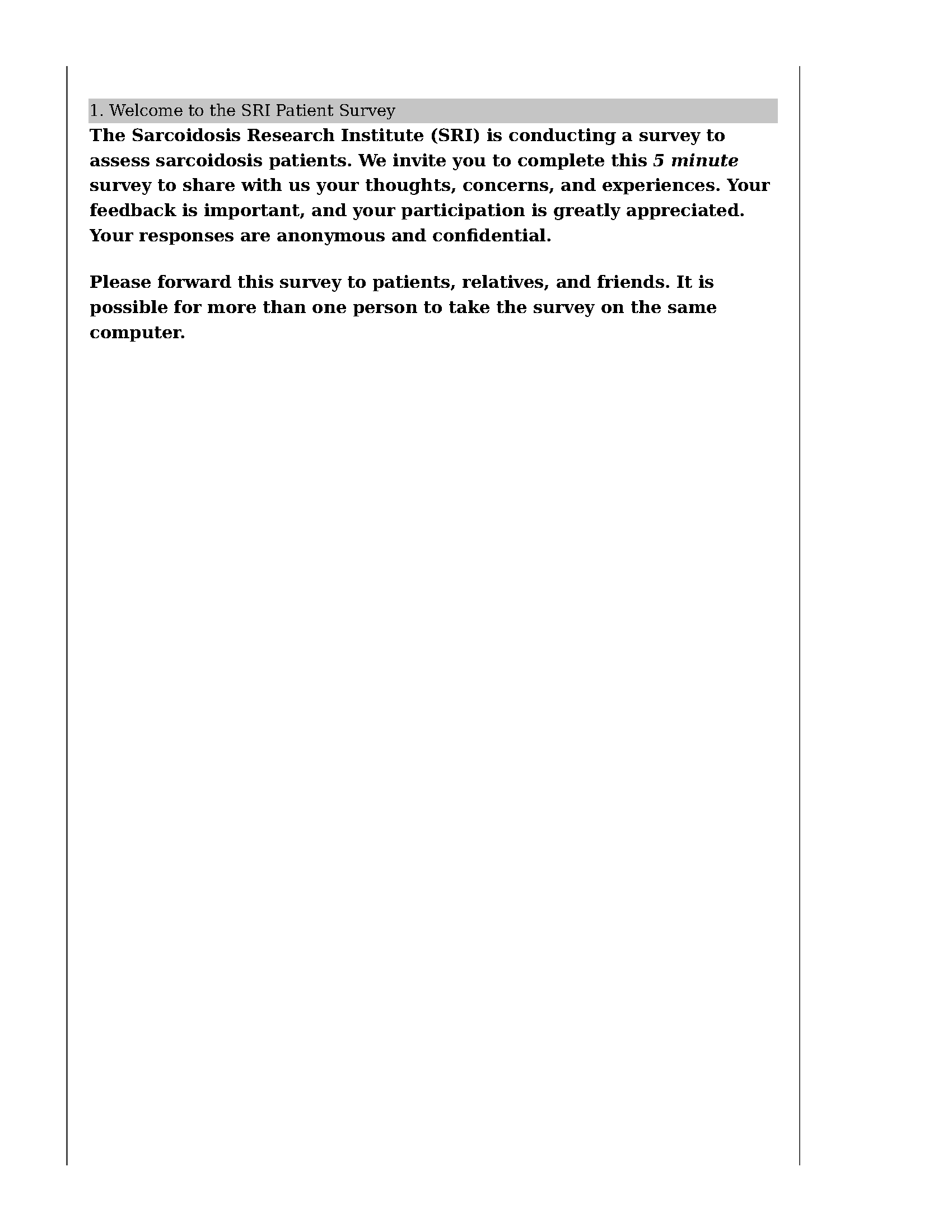

Supplement: Supplementary file 1 — Supplementary file1 (TIFF 101 KB) [file 408_2024_761_MOESM1_ESM.tiff]

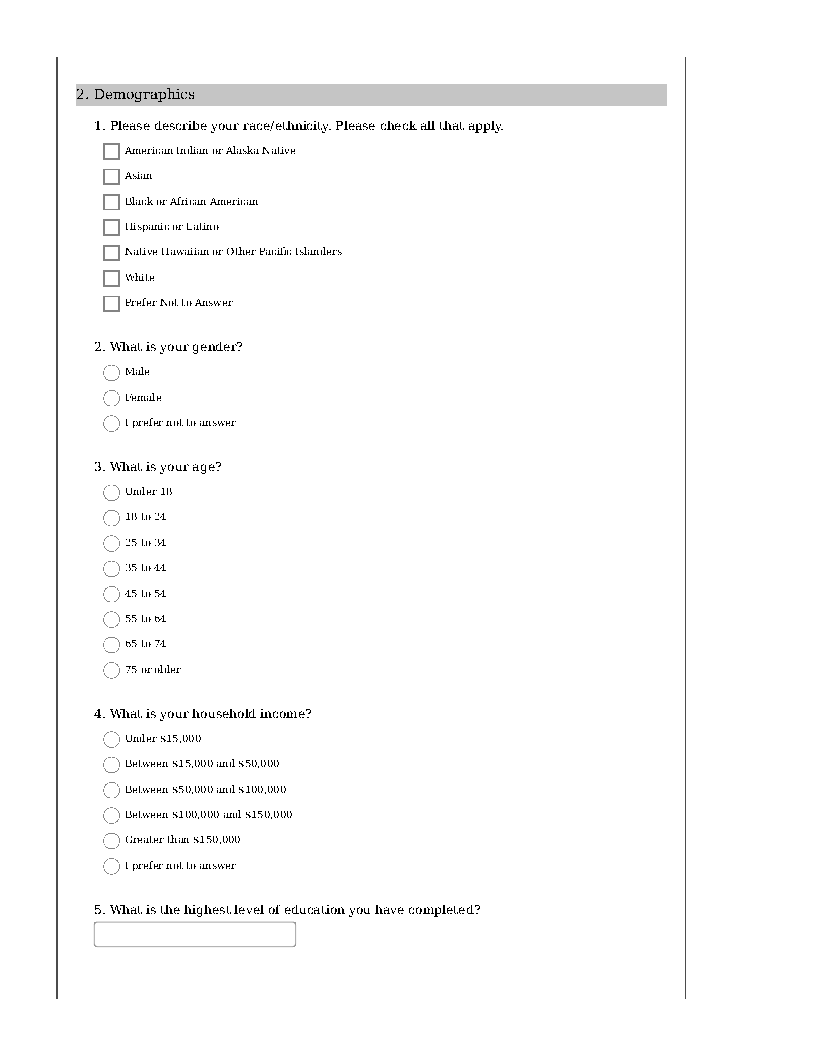

Supplement: Supplementary file 2 — Supplementary file2 (TIFF 26 KB) [file 408_2024_761_MOESM2_ESM.tiff]

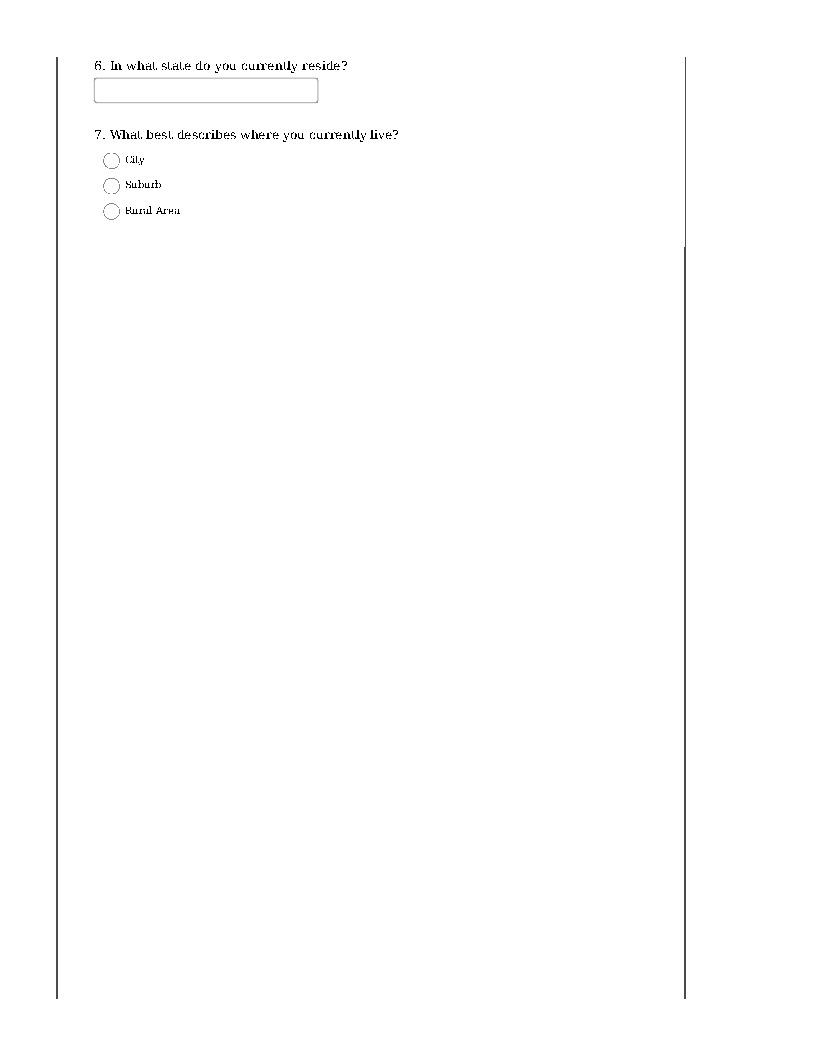

Supplement: Supplementary file 3 — Supplementary file3 (TIFF 21 KB) [file 408_2024_761_MOESM3_ESM.tiff]

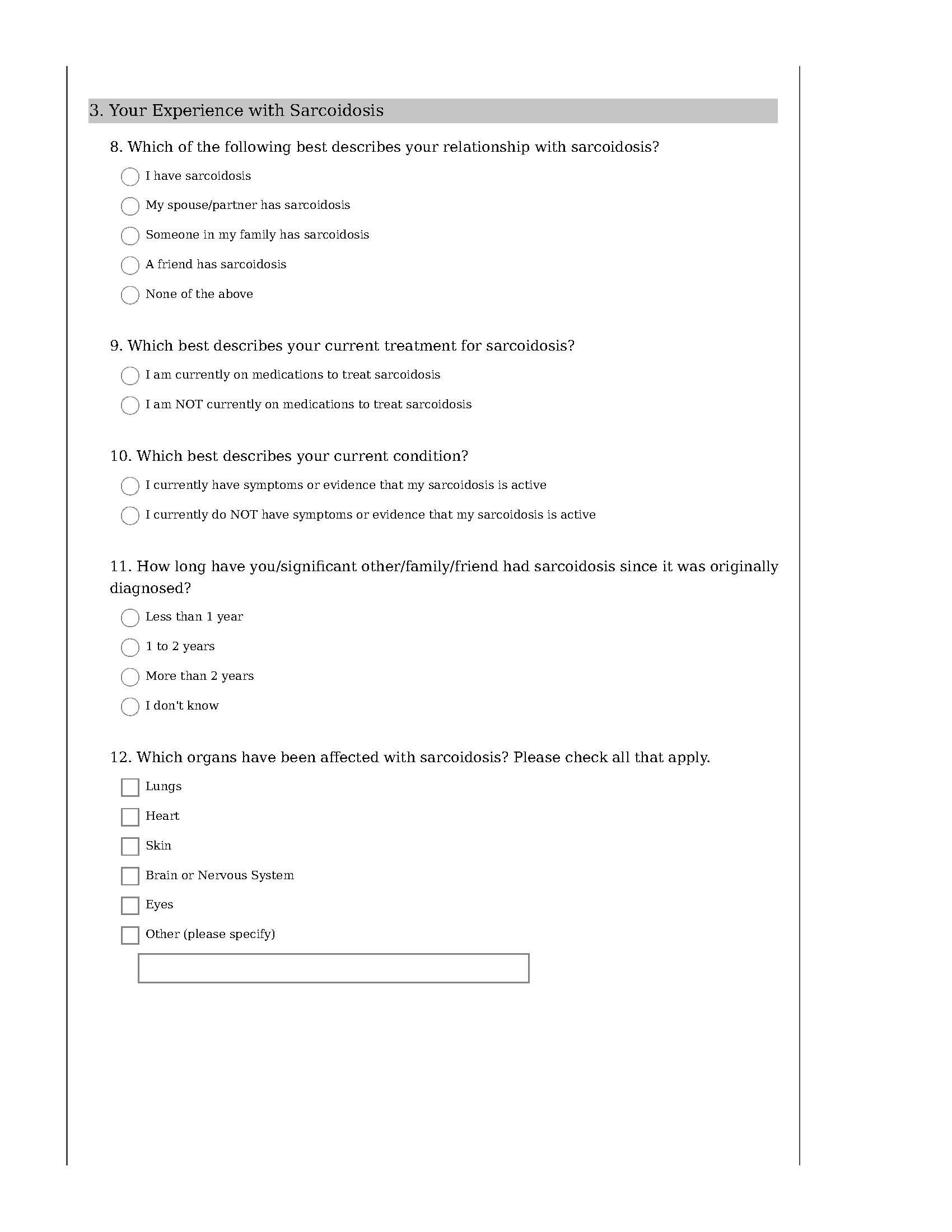

Supplement: Supplementary file 4 — Supplementary file4 (TIFF 109 KB) [file 408_2024_761_MOESM4_ESM.tiff]

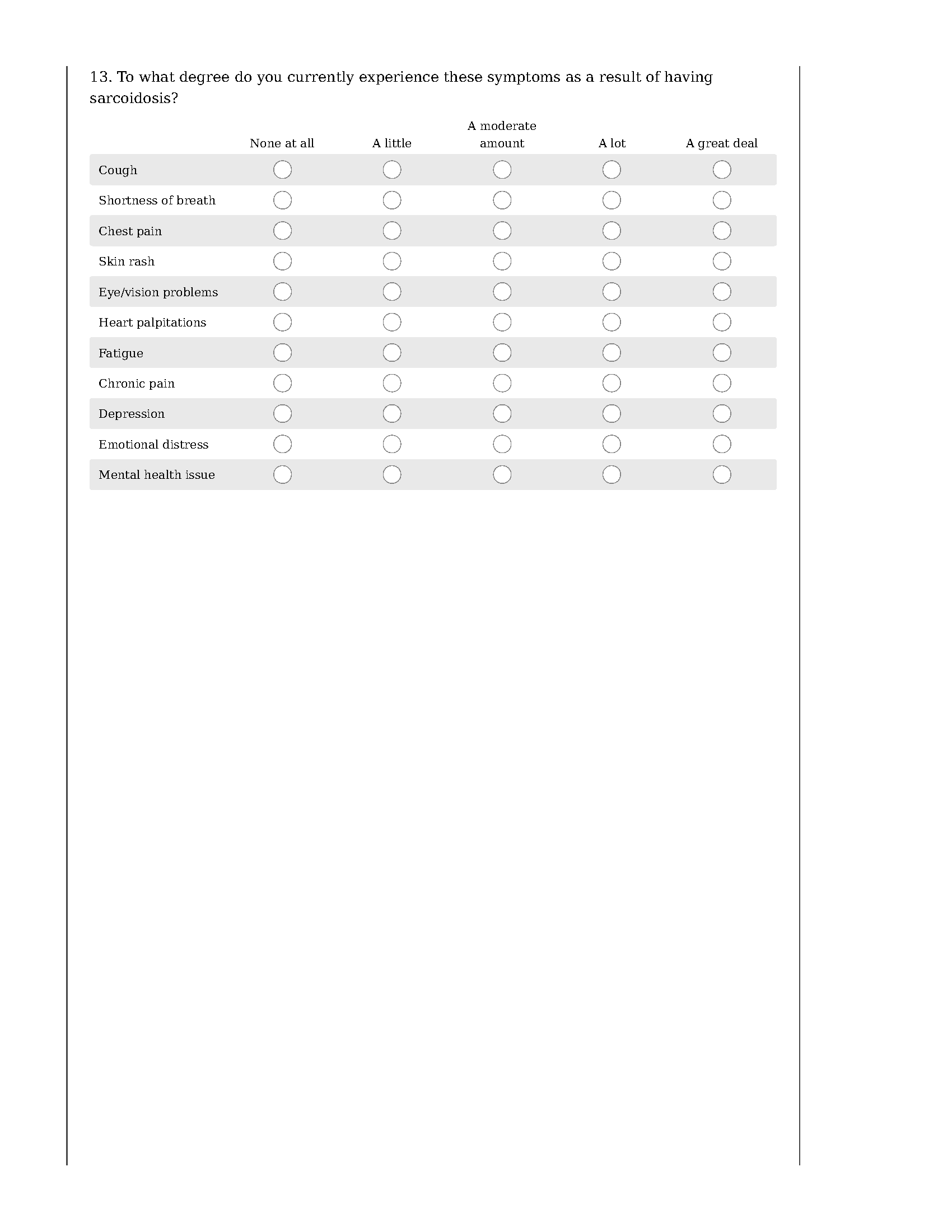

Supplement: Supplementary file 5 — Supplementary file5 (TIFF 103 KB) [file 408_2024_761_MOESM5_ESM.tiff]

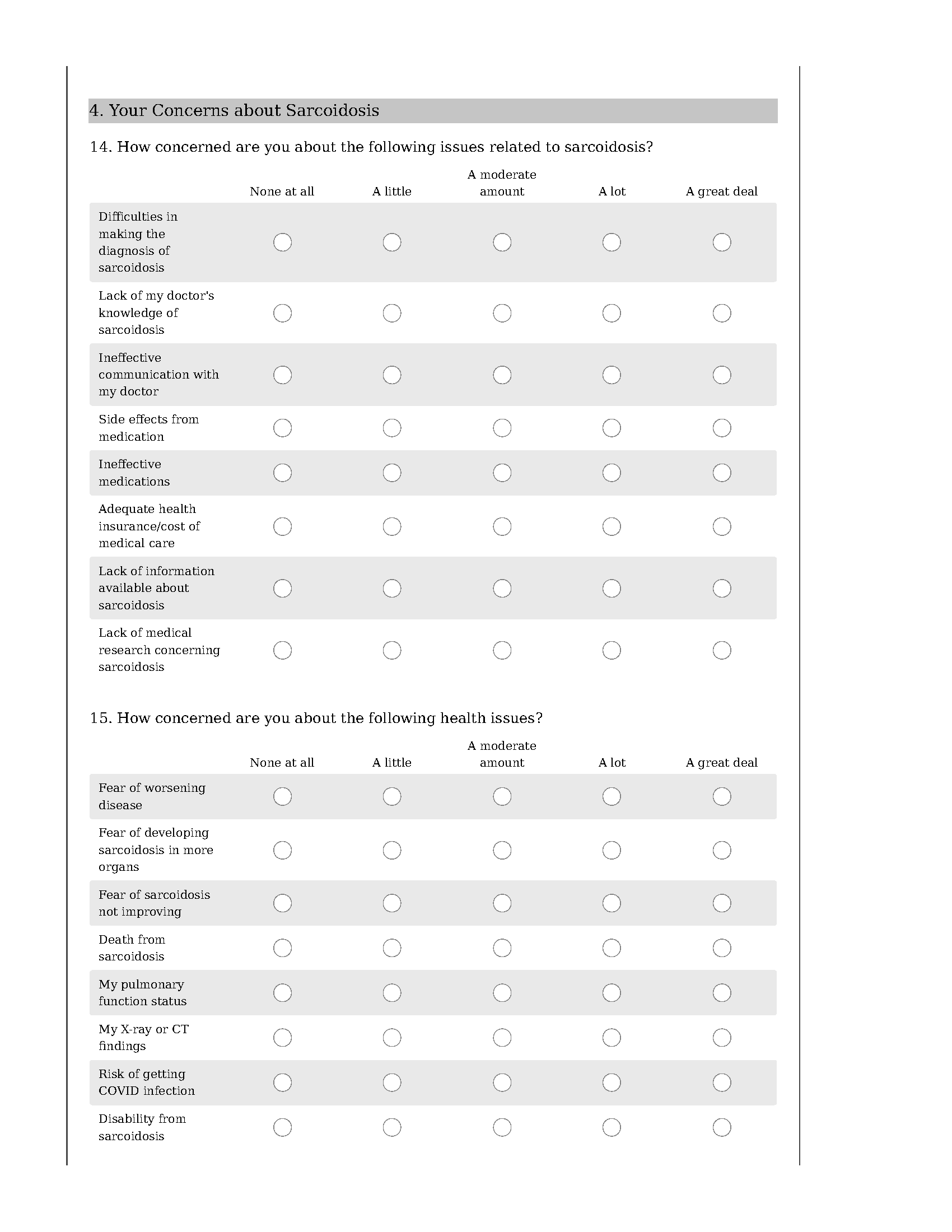

Supplement: Supplementary file 6 — Supplementary file6 (TIFF 125 KB) [file 408_2024_761_MOESM6_ESM.tiff]

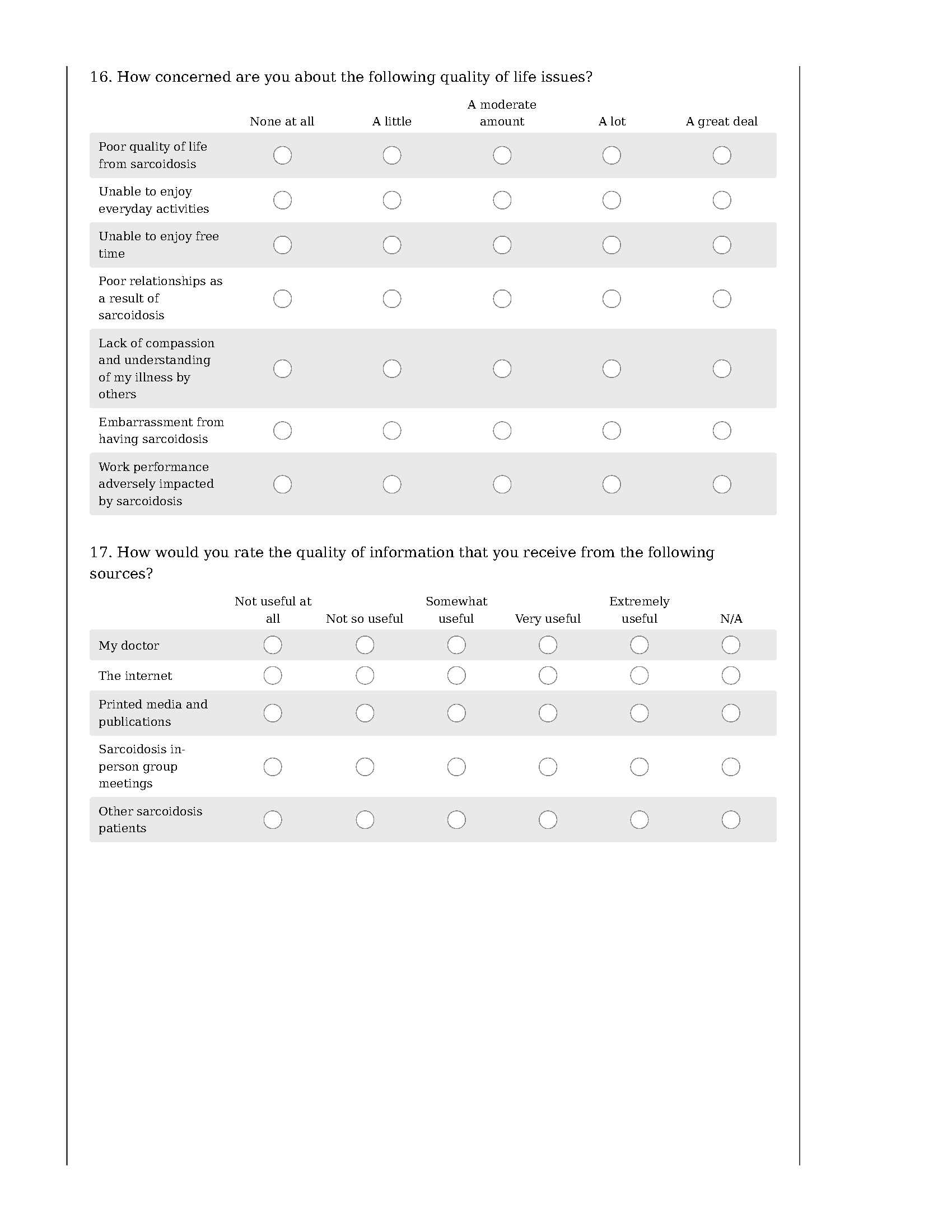

Supplement: Supplementary file 7 — Supplementary file7 (TIFF 117 KB) [file 408_2024_761_MOESM7_ESM.tiff]

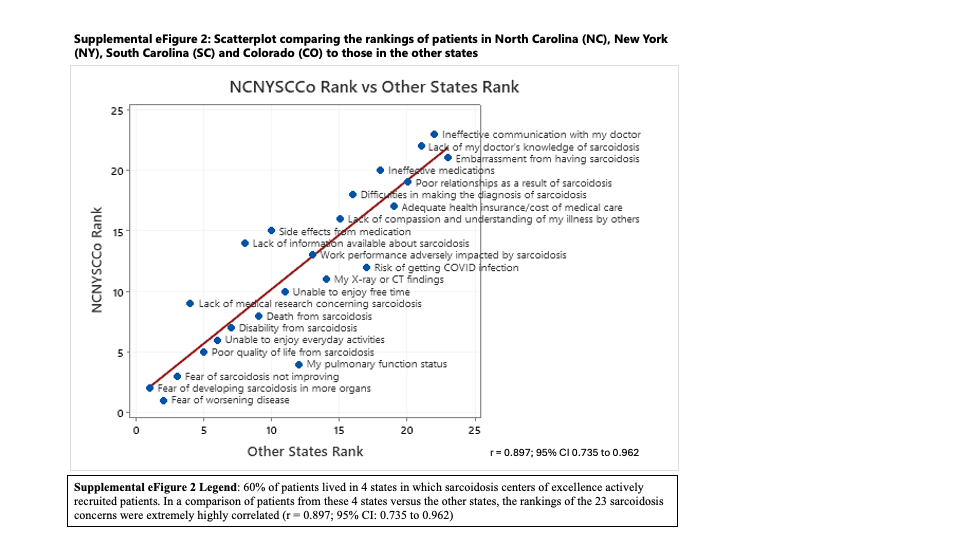

Supplement: Supplementary file 8 — Supplementary file8 (TIFF 1522 KB) [file 408_2024_761_MOESM8_ESM.tiff]
